# Reform in Medical Education

**Published:** 1880-08

**Authors:** 


### EDITORIAL DEPARTMENT.

"If thou hast truth to utter, speak;
And leave the rest to God."

Reform in Medical Education. *Nine Months' Sessions, etc.* We are gratified to learn from intelligent sources that the idea of continuing the sessions in our Medical Colleges for nine months, in order to a thorough presentation and elucidation of the fundamental branches of medical science, and requirement of attendance upon two such sessions at least, before a student should be eligible for the final examination of M. D., has been favorably regarded by gentlemen of experience as teachers in some of our schools. It has been suggested, however, that "four lectures per week, aggregating 144 lectures in obstetrics, would be too many," and that a fewer number would be amply sufficient on that branch, say two at most, as otherwise, the subject could not be extended so far! We are not quite certain that that would inevitably be the case, were *four* lectures delivered each week during a session, or course of nine months. As a former Professor of Obstetrics, we delivered from sixty to seventy lectures, during a five months' session, for several years, and always found our course incomplete at the end of the term. This, however, could not be regarded as an objection to the general proposition for a nine months' term; for should the Professor exhaust the subject of obstetrics, the remaining time of the curriculum could be easily and profitably occupied with lectures on some of the specialties, or in laboratory work and practice; while *all* the other *fundamental branches* would afford abundant material for the "144 lectures," of their respective Professors. One of the great troubles with many of the schools, apart from the irremediable lack of time, is the crowding upon the mind of the student, lectures and demonstrations, on *special* or *collateral* branches, ere he clearly understands the plinths and substrata; or is rendered sufficiently well acquainted with the fundamental departments of the science. If the superstructure is to be embellished and beautified, let it be done *after*, not before, the foundation is secured, and the solid and enduring edifice properly prepared for adornment; otherwise, substantiality, durability, harmony and beauty cannot be expected.

Thus the names, value and relations of letters, are learned but *once*, but always *before* one reads, and is thereby enabled to appreciate the plenitude and sublimities, they are capable of revealing in the physical and in the moral world. The same facts stand good in regard to *everything else* in the universe, as far as we can conjecture, or have any knowledge of them. We, therefore, favor laying the foundations of the radical departments of the profession as thoroughly and securely as possible, and this can only be effected by increase of time, *before* attempting to teach students the special and collateral branches of the profession. Shoemakers, tailors, carpenters, blacksmiths and barbers, even, require at least *three years* apprenticeship in which to learn the principles and practice of their trades; whilst for a decade or more, our doctors of the human body, except in two or three schools, are taught and graduated by ten or a dozen professors, in two short sessions of four to five months, on *all* the accomplishments and facts they can or will receive in that short time, and then turned loose on the public! Yet, the foregoing *are trades*, and our occupation is called "one of the learned profession!" Let us have *real* increase of time for studying the foundations of the science, with all the new specialties, or let us return to the five months' sessions, for teaching *them*, as of yore, and relegate the specialties to the vacations for study and practice.

